# Case Report: Anodal DLPFC-tDCS for refractory post-traumatic cognitive fatigue in the hypometabolic brain

**DOI:** 10.3389/fnhum.2026.1919849

**Published:** 2026-07-31

**Authors:** Erin Glennon, Zhiyan Yang, Joan Stilling

**Affiliations:** 1 Department of Neurology, Weill Cornell Medicine, New York, NY, United States; 2 Department of Rehabilitation Medicine, Weill Cornell Medicine, New York, NY, United States

**Keywords:** dorsolateral prefrontal cortex, fatigue outcomes, home-based brain stimulation, mild traumatic brain injury, neuromodulation, neuroplasticity, post-traumatic cognitive fatigue, transcranial direct current stimulation (tDCS)

## Abstract

**Background:**

Cognitive fatigue is among the most common and disabling long-term sequelae of mild traumatic brain injury (mTBI), with limited effective treatment options. We report a case of refractory post-mTBI cognitive fatigue treated with home-based anodal transcranial direct current stimulation (tDCS) guided by FDG-PET/MRI findings.

**Case presentation:**

A patient sustained an mTBI in 2013 following a high-speed motorcycle accident, with possible loss of consciousness and approximately four hours of post-traumatic amnesia. Despite more than a decade of multidisciplinary rehabilitation, he remained unable to return to work due to severe cognitive fatigue (Fatigue Severity Scale [FSS-7] 5.4/7), post-traumatic headache, fatigue-exacerbated dysarthria, photophobia, phonophobia, and poor sleep (STOP-Bang score: 5, high risk for OSA). At the time of treatment, he was taking methylphenidate and galcanezumab. Pre-treatment FDG-PET/MRI demonstrated bilateral prefrontal hypometabolism (left greater than right) and parietal hypometabolism, with relative hypermetabolism in the left temporal lobe, anterior cingulate cortex, and brainstem. These findings informed selection of the left dorsolateral prefrontal cortex (DLPFC) as the stimulation target.

**Intervention:**

A 14-day home-based tDCS protocol was administered, with the first session completed in clinic. Stimulation was delivered at 2 mA for 20 min daily using an anode placed over F3 (left DLPFC) and cathode over F4 (right DLPFC), concurrent with structured cognitive exercises.

**Outcomes:**

Following treatment, fatigue improved substantially (FSS-7: 5.4 to 3.7), exceeding the reported minimal clinically important difference. Modified Fatigue Impact Score (MFIS: 31 to 27), including the cognitive fatigue subscale (21 to 16). Cognitive performance also improved, with gains in processing speed (Symbol Digit Modalities Test: 46 to 58) and global cognition (Montreal Cognitive Assessment: 26 to 29). Measures of mood, anxiety, and quality of life remained stable. No serious adverse events were reported.

**Conclusion:**

Home-based anodal tDCS targeting the left DLPFC was associated with clinically meaningful reductions in chronic post-mTBI cognitive fatigue and improvements in cognitive performance. This case highlights the potential utility of FDG-PET/MRI as a biomarker to guide personalized neuromodulation and supports further investigation of DLPFC-targeted tDCS for treatment-resistant post-traumatic fatigue.

## 1 Introduction

Up to 40% of patients with mTBI develop persistent post-concussion symptoms (PPCS) beyond three months, with cognitive fatigue among the most disabling (Norrie et al., 2010). Unlike general tiredness, post-mTBI cognitive fatigue is characterized by disproportionate, rapid-onset mental exhaustion with cognitive effort that is not relieved by rest and poorly responsive to current pharmacological and rehabilitative approaches (Johansson et al., 2009). It significantly impairs occupational function, quality of life, and engagement with rehabilitation.

The DLPFC plays a causal role in cognitive effort and fatigue regulation; its disruption increases perceived mental fatigue and impairs effortful task performance (Soutschek and Tobler, 2020; Ishii et al., 2014). Post-TBI disruption of DLPFC connectivity with the ACC, basal ganglia, and thalamus is thought to underlie the characteristic pattern of cognitive exhaustion under load. Analogous prefrontal network dysfunction has been described in MS-related fatigue, where altered ventral striatum-DLPFC resting-state connectivity and maladaptive motor network recruitment over time are established biomarkers (Jaeger et al., 2019; Rocca et al., 2016), providing mechanistic grounds for translating DLPFC-targeted neuromodulation across these conditions.

tDCS modulates cortical excitability via weak direct current delivered through scalp electrodes; anodal stimulation increases neuronal excitability through long term potentiation (LTP)-like mechanisms, while cathodal stimulation has the opposite effect (Nitsche and Paulus, 2000). Anodal tDCS over the left DLPFC has demonstrated efficacy for fatigue in neurological disorders including MS (Jaeger et al., 2019), and home-based tDCS delivery has been shown to be safe, feasible, and well tolerated (Charvet et al., 2018, 2025). Its application specifically for chronic post-mTBI cognitive fatigue in older adults remains understudied.

We report a 73-year-old male with over a decade of post-mTBI cognitive fatigue refractory to multimodal rehabilitation and pharmacotherapy, who underwent a 14-day home-based anodal tDCS protocol targeting the left DLPFC. This case is reported in accordance with the CARE guidelines (Gagnier et al., 2014).

## 2 Case description

### 2.1 Patient history and mechanism of injury

A 73-year-old right-handed male with no prior psychiatric or neurological history presented with chronic post-mTBI cognitive fatigue. In 2013, he sustained an mTBI following a motorcycle accident at approximately 55 mph, with possible loss of consciousness and post-traumatic amnesia of approximately four hours, requiring a two-day hospital admission. He was discharged without surgical intervention. Acute neuroimaging is unavailable; injury classification as mTBI is based on clinical documentation consistent with WHO criteria (GCS 13–15, LOC ≤ 30 min, PTA < 24 h).

### 2.2 Post-injury rehabilitation history

Over more than a decade, the patient engaged in vision therapy, vestibular rehabilitation, and cognitive rehabilitation. Despite sustained participation, cognitive fatigue persisted and was characterized by rapid onset of mental exhaustion with cognitive tasks and slow processing speed. The individual was unable to return to work or meaningful social participation.

### 2.3 Neuroimaging: FDG-PET/MRI findings

Original CT imaging from the acute hospitalization (2013) is unavailable. The patient completed an FDG-PET/MRI prior to the treatment intervention. Approximately 30 min after IV administration of 6.55 mCi F-18 FDG (serum glucose 99 mg/dL), PET imaging was acquired with concurrent MRI for attenuation correction. Structural MRI showed no hydrocephalus, midline shift, stroke, or tumour.

Key metabolic findings included: (1) mild-to-moderate hypometabolism in the superior and middle frontal lobes bilaterally, left greater than right; (2) mixed frontal metabolic imbalance; (3) mild-to-moderate parietal hypometabolism; and (4) mild-to-moderate hypermetabolism in the left temporal lobe, hippocampus, ACC, inferior frontal region, and brainstem. Remaining cortical and subcortical structures were within normal limits.

FDG-PET/MRI source images are unavailable, as the study was conducted at an external facility under a research protocol and de-identified images were not retained for clinical use; the findings reported here are drawn directly from the formal radiology report.

The left frontal lobe, which includes the DLPFC, demonstrated pronounced hypometabolism, confirming the selection of F3 as the anodal stimulation site and providing neuroimaging-guided rationale for the intervention.

### 2.4 Clinical presentation at tDCS assessment

At assessment, the patient reported severe cognitive fatigue (FSS-7: 5.4/7), with a particularly disabling inability to absorb written material. He could read words but could not retain meaning. Fatigue-exacerbated dysarthria was noted clinically under cognitive load. He was not napping despite fatigue severity.

On review of systems, photophobia and phonophobia were present, limiting social and cognitive engagement; urinary urgency was noted; no dysphagia. PHQ-2 screening for depression was 0. A history of post-injury agitation was reported, not active at assessment. Exercise consisted of walking and yardwork. Sleep was consistently poor, with the patient noting approximately five hours on a good night, often restless. STOP-Bang score was 5/8 (snoring, daytime fatigue, hypertension, age >50, and male sex positive), placing him in the high-risk category for obstructive sleep apnoea (OSA). Polysomnography had not been performed prior to tDCS initiation. Post-traumatic headache was constant, predominantly occipital with intermittent bilateral temporal spread, managed with galcanezumab (Emgality). Methylphenidate had been used for cognitive fatigue with partial, inconsistent benefit; modafinil had not been trialed. No sedating medications were in use. Prior to his 2013 injury, the patient had worked in finance; he has not returned to work since.

Baseline neuropsychological assessment demonstrated: MoCA 26/30; SDMT 46; MFIS total 31 (cognitive subscale 21); FAI 34; FSS-7 5.4; PHQ-9 4; GAD-7 2; SF-36 50. Neurological examination was notable for: intact cranial nerves; normal tone and strength; right hand tremor; reduced deep tendon reflexes bilaterally in the lower extremities; and gait imbalance, consistent with his vestibular rehabilitation history. Sensation was intact. No other cerebellar signs were present.

### 2.5 tDCS protocol

Following assessment and written informed consent, a home-based tDCS protocol was initiated. Session 1 was delivered in clinic under direct supervision; sessions 2–14 were self-administered at home following structured training. Protocol parameters: anode F3 (left DLPFC), cathode F4 (right DLPFC), 10–20 EEG system; 2 mA; 30 s ramp up/down; 20 min/session; once daily for 14 consecutive days; concurrent cognitive exercises throughout each session. The bilateral DLPFC montage was selected to enhance left prefrontal excitability consistent with lateralised fatigue regulation and the PET hypometabolism findings (Lefaucheur et al., 2017). Device: Mini-CT tDCS device (Soterix Medical; Woodbridge, NJ). All 14 sessions were completed without significant adverse events; mild transient tingling at electrode sites was the only reported sensation.

### 2.6 Outcome measures

Fatigue severity was assessed using the Fatigue Severity Scale-7 (FSS-7; 7 items; mean item score range 1–7; pathological threshold ≥4.0; minimal clinically important difference [MCID] = 1 point; Learmonth et al., 2013). The FSS-7 is a validated short form of the original Fatigue Severity Scale specifically designed to reduce respondent burden while preserving sensitivity to change.

The multidimensional impact of fatigue was evaluated using the Modified Fatigue Impact Scale (MFIS; Fisk et al., 1994; 21 items; total score range 0–84; three subscales: cognitive [10 items, range 0–40], physical [9 items, range 0–36], and psychosocial [2 items, range 0–8]). Higher scores indicate greater fatigue impact. The MCID for the MFIS total score is ≥4 points in neurological populations (Rooney et al., 2019); the MCID for the cognitive subscale is ≥6.8 points (Kluger et al., 2017).

Global cognition was assessed with the Montreal Cognitive Assessment (MoCA; Nasreddine et al., 2005; 30 items; total score range 0–30; score ≥26 considered within normal limits). The MCID for MoCA in neurological rehabilitation populations is 2–3 points (Wu et al., 2019). Information processing speed was assessed with the Symbol Digit Modalities Test (SDMT; Smith, 1973; number of correct substitutions in 90 s; higher scores indicate better performance; normative values are age- and education-adjusted). The MCID for SDMT in neurological populations is 4–5 points (Benedict et al., 2017).

Depression was screened using the Patient Health Questionnaire-9 (PHQ-9; Kroenke et al., 2001; 9 items; total score range 0–27; scores of 0–4 = minimal, 5–9 = mild, 10–14 = moderate, 15–19 = moderately severe, 20–27 = severe depression). The MCID is 5 points (Löwe et al., 2004). Anxiety was assessed using the Generalised Anxiety Disorder Scale-7 (GAD-7; Spitzer et al., 2006; 7 items; total score range 0–21; score ≥10 indicates clinically significant anxiety). The MCID is 4 points (Toussaint et al., 2020).

Health-related quality of life was assessed using the Short Form-36 Health Survey (SF-36; Ware et al., 1993; 36 items; scores range 0–100 per domain; higher scores indicate better health-related quality of life). The MCID ranges from 5 to 10 points depending on domain and population (Ware et al., 1993).

Real-world activity participation was assessed using the Frenchay Activities Index (FAI; Holbrook and Skilbeck, 1983; 15 items assessing domestic, leisure, and outdoor activities over a defined recall period; total score range 0–45; higher scores indicate greater activity participation). The FAI was originally developed and validated in stroke rehabilitation populations. No formally established MCID specific to mild traumatic brain injury populations has been confirmed in the published literature; FAI data are therefore reported as directional only and interpreted with caution. Importantly, higher FAI scores indicate greater activity participation (better function); declining scores represent reduced participation.

All instruments were administered at four timepoints: baseline (pre-tDCS), during tDCS (mid-protocol, day 7–8), post-tDCS (within one week of completing the 14-day protocol), and one-month follow-up. MoCA and SDMT were administered at baseline and post-tDCS only and were not repeated at one-month follow-up.

### 2.7 Episode of care timeline

Table 1 presents the key clinical events and outcome measures across the patient’s episode of care, from injury to post-tDCS follow-up assessment.

**Table 1:** Episode of care timeline.

Timepoint	Event/Intervention	FSS-7 score	Key finding/Status
2013—Injury	Motorcycle accident ~55 mph; possible LOC; PTA ~ 4 h; hospitalized 2 days	-	mTBI diagnosis; discharged to outpatient rehab
2013—ongoing	Vision therapy, vestibular rehab, cognitive therapy	-	Partial functional recovery; fatigue persists
Pre-tDCS Baseline	Presenting for tDCS trial; on Ritalin; chronic occipital headache (Emgality); 5 h sleep/night	5.4/7	FSS-7 above clinical threshold; MFIS = 31; MoCA = 26; SDMT = 46
During tDCS (Days 1–14)	First session in clinic; 13 sessions home-based. Anodal tDCS: L-DLPFC (F3), 2 mA, 20 min/day + concurrent cognitive exercises	4.6/7	MFIS = 22; MFI Cog = 14; FAI = 27; good tolerability
Post-tDCS	Follow-up assessment; no change to medications	3.7/7	FSS-7 below MCID threshold; MoCA = 29 (+3); SDMT = 58 (+12); MFIS = 27; FAI = 32

FSS-7, Fatigue Severity Scale-7 (score range 1–7; ≥4.0 indicates clinically significant fatigue); LOC, loss of consciousness; PTA, post-traumatic amnesia; mTBI, mild traumatic brain injury; MFIS, Modified Fatigue Impact Score; MoCA, Montreal Cognitive Assessment; SDMT, Symbol Digit Modalities Test; FAI, Frenchay Activities Inventory; L-DLPFC, left dorsolateral prefrontal cortex.

## 3 Results

### 3.1 Neuroimaging basis for treatment selection

Pre-treatment FDG-PET/MRI informed stimulation target selection. Left-predominant hypometabolism in the superior and middle frontal lobes localized the metabolic deficit to the DLPFC network, which can be implicated in cognitive effort and fatigue regulation (Soutschek and Tobler, 2020; Ishii et al., 2014). The pattern of reduced prefrontal metabolism paired with compensatory ACC and brainstem hypermetabolism is consistent with the cortico-reticular fatigue model: a hypoactivated top-down control system driving disproportionate arousal-circuit engagement under cognitive load. Unlike prior tDCS reports in TBI populations where target selection was based on clinical phenotype alone, this case demonstrates neuroimaging-guided neuromodulation.

### 3.2 Quantitative outcome measures

Table 2 summarizes outcomes across all timepoints. Asterisks (*) denote changes meeting or exceeding the established minimal clinically important difference (MCID).

**Table 2:** Clinical outcome measures.

Outcome measure	Baseline	During tDCS	Post-tDCS	1-month follow-up	MCID
FSS-7 (fatigue severity)	5.4	4.6	3.7*	4.9	1 point (Learmonth et al., 2013)
MFIS (modified fatigue impact scale)	31	22	27*	23*	4 points (Rooney et al., 2019)
MFIS cognitive subscale	21	14	16*	13*	6.8 points (Kluger et al., 2017)
SF-36 (quality of life)	50	50	50	50	5–10 points (Ware et al., 1993)
PHQ-9 (depression)	4	7	5	5	5 points (Löwe et al., 2004)
GAD-7 (anxiety)	2	2	2	4	4 points (Toussaint et al., 2020)
MoCA (global cognition)	26	—	29*	—	2–3 points (Wu et al., 2019)
SDMT (processing speed)	46	—	58*	—	4–5 points (Benedict et al., 2017)
FAI (functional activities)	34	27	32	29	

*change meets or exceeds MCID. MCID, minimal clinically important difference. MoCA and SDMT were not re-administered at 1-month follow-up.

**Figure 1:**
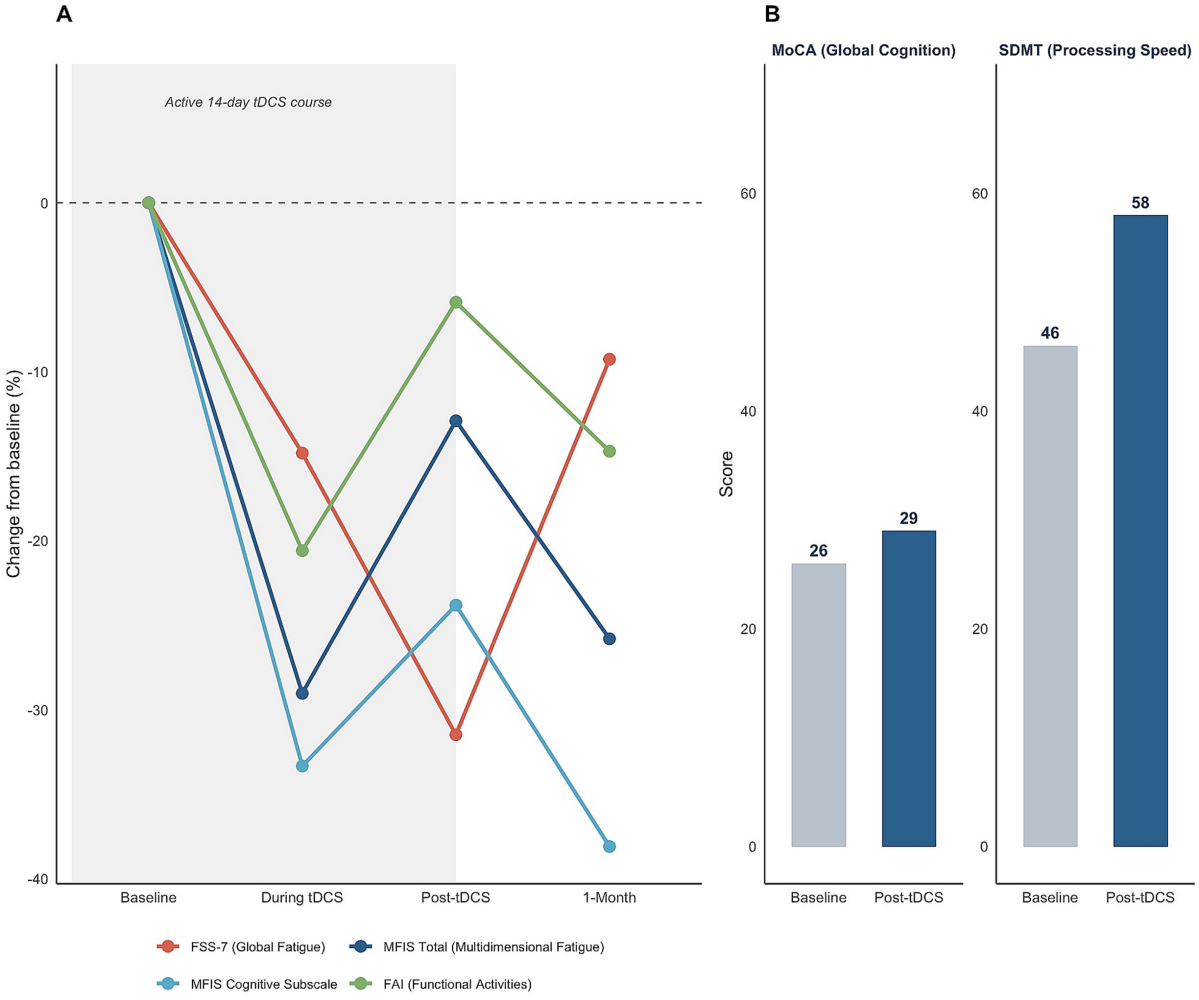
Fatigue and cognitive outcomes following 14-day anodal tDCS over the left DLPFC. **(A)** Percent change from baseline in fatigue and functional activity measures across the treatment course (Baseline, During tDCS, Post-tDCS, and 1-Month follow-up). The shaded region indicates the active 14-day stimulation period. FSS-7 (global fatigue) and MFIS total (multidimensional fatigue) improved progressively through the post-tDCS assessment before partially rebounding at one month; in contrast, the MFI cognitive subscale (functional activities) showed continued or sustained improvement at one month relative to post-tDCS. **(B)** Global cognition (MoCA) and processing speed (SDMT) at baseline and post-tDCS, both showing improvements exceeding their respective minimal clinically important differences. Cognitive measures were not re-administered at one-month follow-up. FSS-7, fatigue severity scale-7; MFIS, modified fatigue impact scale; FAI, Frenchay activities inventory; MoCA, Montreal cognitive assessment; SDMT, symbol digit modalities test; tDCS, transcranial direct current stimulation; DLPFC, dorsolateral prefrontal cortex.

FSS-7 improved progressively from 5.4 at baseline to 4.6 during treatment and 3.7 post-tDCS—a 1.7-point reduction exceeding the MCID of 1 point and crossing below the pathological threshold of 4.0. At one-month follow-up, however, FSS-7 partially rebounded to 4.9, returning above threshold. This pattern of robust immediate improvement with partial regression at one month suggests that a single 14-day course may produce time-limited relief and raises the question of whether booster sessions or longer protocols are needed to sustain benefit (Figure 1).

In contrast, MFIS continued to improve at one month (31 → 27 post-tDCS → 23 at one month; cumulative reduction of 8 points, well above the MCID of 1–2 points), as did the cognitive fatigue subscale (21 → 16 → 13). This dissociation between FSS-7 and MFIS at one month is noteworthy: the FSS-7 captures global fatigue severity, while the MFIS differentiates cognitive, physical, and psychosocial dimensions. Continued MFIS improvement alongside FSS-7 rebound may indicate that cognitive fatigue and activity engagement continued to benefit from residual neuroplastic effects, even as global fatigue severity returned toward threshold.

MoCA improved from 26 to 29 post-tDCS (exceeding the 2–3 point MCID), and SDMT from 46 to 58 (a 12-point gain, more than double the MCID of 4–5 points). The SDMT improvement is particularly notable given its sensitivity to post-TBI white matter disruption and DLPFC-mediated processing speed. Neither was re-assessed at one month, which limits conclusions about cognitive durability.

FAI declined across all post-baseline timepoints (34 → 27 during → 32 post-tDCS → 29 at one month). SF-36 remained stable at 50; PHQ-9 was stable at 4–5, with no mood deterioration. GAD-7 rose from 2 to 4 at one month, below clinical threshold and not meeting the MCID of 4 points. The intervention was well tolerated throughout.

## 4 Discussion

### 4.1 Strengths and clinical significance

Several features of this case strengthen its scientific contribution. First, pre-treatment FDG-PET/MRI supported the tDCS stimulation target, with left frontal hypometabolism corresponding to the F3 anode site. Neuroimaging-guided target selection is uncommon in tDCS interventions to date. Second, the patient’s age (73 years) and symptom chronicity (>10 years post-injury) extend the evidence base for DLPFC tDCS beyond the younger, sub-acute populations studied in most trials. Third, the predominantly home-based delivery model (one supervised clinic session; 13 home sessions) demonstrates practical feasibility in a fatigued, older population. This is a meaningful clinical consideration given the access barriers this group faces.

Concurrent cognitive exercise during stimulation is mechanistically important. Hebbian co-activation principles predict more robust and durable synaptic potentiation when increased cortical excitability (tDCS) coincides with active engagement of the targeted network (Vergallito et al., 2023; Li et al., 2019). *In vitro* rat hippocampal slice physiology work showed that DCS enhanced LTP only at synapses undergoing plasticity, further supporting this mechanism in tDCS (Kronberg et al., 2020). The specificity of improvement—most pronounced in cognitive fatigue and processing speed, with preserved mood and quality of life—is consistent with DLPFC-mediated top-down fatigue modulation rather than a non-specific activating effect.

### 4.2 Limitations

This is a single uncontrolled case; expectation effects and concurrent cognitive exercise cannot be excluded as contributors to treatment response. Continued methylphenidate use throughout the protocol represents a pharmacological confound, though the longstanding and partial nature of stimulant benefit prior to tDCS makes it an unlikely sole explanation for the observed gains.

The partial rebound in FSS-7 score at one-month follow-up (3.7 → 4.9) is an important limitation with respect to treatment durability. Whether this reflects waning of tDCS associated neuroplastic effects, natural fluctuation in fatigue severity, or factors unrelated to the intervention (such as changes in physical activity, sleep, or headache burden) cannot be determined from a single uncontrolled case. The divergence between FSS-7 rebound and continued MFIS improvement at one month further illustrates the complexity of fatigue trajectory assessment and the importance of using multiple instruments rather than relying on a single primary endpoint.

FDG-PET images were obtained from an external research protocol and de-identified images were not retained. The findings were drawn from the formal radiology report alone. Further, FDG-PET demonstrated hypometabolism in both DLPFC regions, yet the protocol applied cathodal (inhibitory) stimulation to the right DLPFC (F4) which is theoretically counterproductive given its hypometabolic state, and potentially attenuating the treatment effect. A more neuroimaging-consistent approach would place the cathode at an extracephalic or supraorbital site (Fp2), allowing bilateral anodal excitation of both hypometabolic regions. Future trials in patients with bilateral prefrontal hypometabolism should consider this modification.

The STOP-Bang score of 5 is a clinically important finding. Undiagnosed OSA is an independent driver of cognitive fatigue, non-restorative sleep, and impaired concentration. Formal polysomnography should be completed, as OSA treatment may independently or additively improve fatigue outcomes. However, this is a chronic symptom that was stable throughout the observed period. More broadly, this patient’s complex post-mTBI syndrome characterized by headache, tremor, gait imbalance, photophobia, phonophobia, and poor sleep reflects multiple interacting contributors to fatigue that cannot be disentangled in an uncontrolled case. MoCA and SDMT were not repeated at one month, limiting conclusions about cognitive durability. Home-based electrode placement introduces a potential consistency confound, though the patient’s training and consistent stimulation sensations across sessions are reassuring.

No post-treatment neuroimaging was performed, so this case cannot speak to whether tDCS altered the underlying metabolic deficit. This is consistent with the literature, where single-session tDCS has not been shown to produce immediate changes in cerebral glucose metabolism (Kraus et al., 2019), whereas repeated daily stimulation over weeks to months using the same F3/F4, 2 mA montage applied here has been associated with measurable shifts in regional glucose metabolism alongside cognitive improvement (Im et al., 2019). Pairing clinical outcomes with follow-up FDG-PET would help establish whether symptomatic improvement after repeated tDCS tracks with normalization of frontal hypometabolism.

Additionally, the absence of acute imaging prevents characterization of the original injury pattern and therefore limits our ability to directly link the current FDG-PET hypometabolism to a specific traumatic mechanism. The 10-year symptom continuity from the time of injury, absence of any intercurrent neurological diagnosis, and consistency between the imaging phenotype and clinical presentation are the available evidence supporting TBI as the etiology, but we acknowledge this cannot be established with certainty.

Finally, the possibility of a neurodegenerative process (Alzheimer’s disease, CTE) contributing to the frontal hypometabolism warrants acknowledgment. However, symptoms were continuous from the time of injury without progressive decline, cognitive impairment was borderline and partially reversible (MoCA 26 → 29), and mood and behavioural screening were normal. These are features more consistent with post-traumatic sequelae than neurodegeneration. This differential cannot be formally excluded without amyloid PET or CSF biomarker evaluation, which is recommended in future follow-up.

### 4.3 Mechanistic considerations

The mechanistic rationale for this case is supported by neuroimaging. Left-predominant DLPFC hypometabolism on FDG-PET corresponds to the neural substrate that Soutschek and Tobler (2020) identified as causally necessary for effortful cognitive engagement. Its disruption produces the clinical phenotype of rapid fatigue onset under cognitive load observed in our patient. Concurrent ACC and brainstem hypermetabolism is consistent with compensatory cingulate-arousal upregulation in the setting of reduced prefrontal control, a metabolically costly strategy that may itself amplify fatigue burden. Anodal tDCS is hypothesized to restore excitability to the hypoactivated DLPFC through LTP-like mechanisms, reducing the neural cost of cognitive effort and recalibrating the DLPFC-ACC-striatal circuit toward greater effort tolerance (Nitsche and Paulus, 2000; Chaudhuri and Behan, 2004). Whether such excitability changes translate into measurable shifts in regional glucose metabolism likely depends on treatment duration. For example, a 6-month at-home tDCS trial in Alzheimer’s disease using the same F3/F4, 2 mA montage reported improved cognition alongside changes in regional cerebral glucose metabolism (Im et al., 2019), whereas single-session protocols have not shown acute metabolic change (Kraus et al., 2019). This suggests that the 14-day course studied here may be sufficient to engage neuroplastic mechanisms clinically without yet producing a metabolic signature detectable on imaging. These mechanisms parallel those proposed in MS-related fatigue, where DLPFC-ventral striatum dysconnectivity and maladaptive network recruitment are established biomarkers (Jaeger et al., 2019; Rocca et al., 2016).

Anodal tDCS over the left DLPFC is hypothesized to restore a degree of cortical excitability to a hypoactivated network, reducing the effort cost of cognitive operations and attenuating fatigue onset. The DLPFC-ACC-striatal circuit is additionally implicated in the motivational and effort-based decision-making aspects of fatigue, where there is tendency to disengage from effortful tasks when perceived cost exceeds expected benefit (Chaudhuri and Behan, 2004). By enhancing DLPFC excitability, tDCS may recalibrate this circuit toward greater effort tolerance and reduced avoidance of cognitive engagement. These mechanisms are supported by neuroimaging studies in MS-related fatigue showing abnormal ventral striatum-DLPFC connectivity (Jaeger et al., 2019) and maladaptive motor network recruitment under fatigue conditions (Rocca et al., 2016), which may have analogous post-TBI counterparts. Further, in healthy participants, PET imaging shows that tDCS to the DLPFC increases endogenous release of dopamine in the ventral striatum with concomitant improvement in attention and working memory (Fukai et al., 2019). An EEG study in mTBI patients suggest that the connectivity and neuroplasticity within the DLPFC is disrupted (Moore et al., 2026). Together, these data support further investigation of tDCS to enhance the reduced neuroplasticity in the DLPFC of mTBI patients.

### 4.4 Clinical implications

Several important clinical implications emerge from this case. Symptom chronicity should not prevent consideration for neuromodulation interventions, as this patient achieved clinically meaningful gains more than a decade post-injury. A 14-day home-based protocol with a single supervised session is feasible and well tolerated in older adults with significant fatigue burden. Concurrent cognitive exercise should be a standard component of tDCS protocols to optimize activity-dependent plasticity. The partial FSS-7 rebound at one month alongside sustained MFIS gains suggests that booster sessions or longer initial courses may be needed for durable remission, and that a multidimensional fatigue battery captures treatment response more comprehensively than a single scale. OSA screening with STOP-Bang should be routine in this population. A score ≥5 warrants polysomnography before and after tDCS to characterize its independent contribution to fatigue and potential additive effects of treatment. As a single uncontrolled case, this report cannot establish causal efficacy of tDCS for post-mTBI cognitive fatigue. Rather, it provides a neuroimaging-supported hypothesis sufficiently specific to motivate a controlled trial.

## 5 Patient perspective

Cognitive fatigue, for me, is feeling like you worked a 20-h day, but in 1 h. I would make mistakes, could not recall things, my vision became blurry because I was cross-eyed. At its worst, I actually would write words upside down and backwards. Having your brain not able to function after such a short time is also emotionally exhausting.

The sessions themselves were quite simple. I did vision therapy and some reading during the sessions, which are activities that fatigue my brain quickly. Sessions are painless, with the only effect on me being that I was tired afterwards. I had to live in New York City, away from my quiet, rural neighborhood, during the treatment, so I cannot make a direct comparison to effects from outside influences and the treatment. However, I feel that I was tolerating the additional noises and movement better, feeling less fatigued and affected by them. I was able to read a book and remember it even after reading for more than an hour.

After returning home, over time, the time it took to get fatigued grew shorter again, until there seemed to be no difference compared to before treatment. Overall, I feel there is great potential for this treatment. Maybe a longer time doing it, or pinpointing the damaged areas of the brain, would make it last longer. Possibly a home unit to keep the healing activity going in between doctor visits. I definitely liked that it is a non-invasive procedure.

## Data Availability

The original contributions presented in the study are included in the article/supplementary material, further inquiries can be directed to the corresponding author.
